# Correction: Bishayee et al. Lotus (*Nelumbo nucifera* Gaertn.) and Its Bioactive Phytocompounds: A Tribute to Cancer Prevention and Intervention. *Cancers* 2022, *14*, 529

**DOI:** 10.3390/cancers14092116

**Published:** 2022-04-24

**Authors:** Anupam Bishayee, Palak A. Patel, Priya Sharma, Shivani Thoutireddy, Niranjan Das

**Affiliations:** 1 College of Osteopathic Medicine, Lake Erie College of Osteopathic Medicine, Bradenton, FL 34211, USA; ppatel24886@med.lecom.edu (P.A.P.); PSharma44656@med.lecom.edu (P.S.); SThoutired89922@med.lecom.edu (S.T.); 2 Department of Chemistry, Iswar Chandra Vidyasagar College, Belonia 799155, Tripura, India; ndnsmu@gmail.com

## Table Legend

In the original article, there was a mistake in the legend for Table 2 [1]. In the table legend (page 24), “in vivo” appears incorrectly which should be replaced by “in vitro”. The correct legend appears below.

**Table 2.** Potential anticancer effects and mechanisms of action of *N. nucifera*-derived constituents based on in vitro studies.

## Error in Table

In the original article, there were mistakes in Table 3 as published. In the table legend (page 32), “in vitro” appears incorrectly which should be replaced by “in vivo”. Additionally, the content of the table is same as Table 2, which should be replaced by a correct table. The corrected Table 3 along with the title appear below.

**Table 3:** Potential anticancer effects and mechanisms of action of *N. nucifera*-derived constituents based on in vivo studies.

Materials Tested	Animal Tumor Models	Anticancer Effects	Mechanisms	Dose (Route)	Duration	References
*Breast cancer*						
Flavonoid-rich leaf extract	BALB/c athymic nude mice injected with MCF-7 cells	Reduced tumor volume and weight	↓HER2; p-HER2; ↓Fas	0.5 & 1% (diet)	28 days	Yang et al., 2011 [79]
Aqueous leaf extract	MDA-MB-231 cells injected in female C57BL/6 nude mice	Inhibited tumor growth	Not reported	0.5–2 % (s.c.)	14 days	Chang et al., 2016 [80]
Liensinine + doxorubicin	Female nude mice injected with MDA-MB-231 cells	Reduced tumor growth	↑Apoptosis; ↑cleaved caspase-3; ↓autophagy/mitophagy; ↑auto-phagosome /mitophagosome; ↑colocalization of DNM1L and TOMM20	60 mg/kg (i.p.); 2 mg/kg (i.p.)	30 days	Zhou et al., 2015 [90]
*Colon cancer*						
Nuciferine	CT29 cells subcutaneously implanted in nude mice	Reduced tumor weight	Not reported	9.5 mg/kg (i.p.)	3 times a week for 3 weeks	Qi et al., 2016 [96]
Liensinine	HT29 cells injected in female BALB/c nude mice	Suppressed colorectal tumorigenesis, reduced tumor size	↓Ki-67	30 mg/kg (oral)	Every other day for 15 days	Wang et al., 2018 [97]
*Eye cancer*						
Neferine	WERI-Rb-1 cells injected in female athymic nude mice	Reduced tumor volume and weight	↓Ki-67; ↓VEGF; ↓SOD; ↑MDA	0.5–2 mg/kg (i.p)	Every 3 days for 30 days	Wang et al., 2020 [100]
*Gallbladder cancer*						
Liensinine	NOZ cells injected in BALB/c nude mice	Reduced tumor volume and weight	↓Ki-67	2 mg/kg (i.p)	Every 2 days	Shen et al., 2019 [101]
*Gastric cancer*						
Liensinine from seeds	SGC7901 cells injected in BALB/c homozygous (nu/nu) nude mice	Reduced tumor size	↓Ki-67	10 µM (i.p.)	Every 2 days for a month	Yang et al., 2019 [106]
*Head and neck cancers*						
Neferine	CAL27 cells injected in male BALB/c nude mice	Reduced tumor volume	↑Apoptosis; ↑autophagy, ↑cleaved caspase-3, ↑cleaved PARP1, ↑LC3; ↑p62	10 mg/kg (i.p)	Not reported	Zhu et al., 2021 [107]
*Liver cancer*						
Water-soluble polysaccharides from seeds	H22 cells injected in female Kunming mice	Reduced tumor weight	↑TNF-ɑ; ↑IL-2; ↑SOD; ↓MDA	50–200 mg/kg (oral)	14 days	Zheng et al., 2016 [102]
Leaf extract	DEN fed male Sprague-Dawley rats	Reduced tumor size	↓AST; ↓ALT; ↓albumin; ↓total triglyceride; ↓total cholesterol; ↓lipid peroxidation; ↑GSH; ↑GSHPx; ↑SOD; ↑CAT; ↑GST; ↓Rac1; ↓PKCɑ; ↓TNF-ɑ; ↓IL-6	0.5–2.0% (p.o.)	12 weeks	Horng et al., 2017 [119]
Leaf extract	2-AAF-induced male Wistar rats	Inhibited hepatic fibrosis and hepatocarcinogenesis	↓Triglycerides; ↓total cholesterol; ↓AFP; ↓IL-6; ↓TNF-ɑ; ↓AST; ↓ALT; ↓γGT; ↓GST-Pi; ↓lipid peroxidation; ↓8-OHdG; ↑Nrf2; ↑CAT; ↑GPx; ↑SOD-1	0.5–2% in the diet (p.o.)	6 months	Yang et al., 2019 [120]
Neferine+oxaliplatin	HepG2 and Bel-7402 cells injected in male BALB/c mice	Increased tumor volume reducing the effect of oxaliplatin	↑E-cadherin; ↓Vimentin; ↓Ki-67;	20 mg/kg/d (i.p.)	3 weeks	Deng et al., 2017 [116]
Isoliensinine	Huh-7 cells injected in male athymic nude mice and H22 cells injected in Kunming mice	Reduced tumor volume	↑caspase-3; ↓Bcl-2; ↓Bcl-xL; ↓MMP-9; ↓p65 phosphorylation	3 and 10 mg/kg/d (i.p. and gavage)	10 days; 3 weeks	Shu et al., 2015 [117]
Isoliensinine	Huh-7 cells transfectants injected in male athymic nude mice	Reduced tumor growth	↑Caspase-3 activity	10 mg/kg/d (gavage)	20 days	Shu et al., 2016 [118]
*Lung cancer*						
Leaf extract and leaf polyphenol extract	4T-1 metastatic tumor in the lung of BALB/c mice	Reduced metastasis and tumor weight	↓PKCɑ activation	0.25, 1% (p.o.)	19 days	Wu et al., 2017 [81]
Nuciferine	A549 cells injected in BALB/c mice	Reduced tumor size and weight	↑Apoptosis; ↓Bcl-2; ↑Bax; ↓Wnt/*β*-catenin; ↑Axin	50 mg/kg (i.p.)	3 times a week for 20 days	Liu et al., 2015 [126]
Neferine	DEN-induced lung carcinogenesis in albino male Wistar rats	Suppressed tumor growth	↓ROS; ↓lipid peroxidation; ↓protein carbonyl; ↑GSH; ↑SOD; ↑GPx; ↑GST; ↑CAT; ↓glycoprotein components; ↑ATPase; ↑p53; ↑Bax; ↑caspase-9; ↑caspase-3; ↓Bcl-2; ↓COX-2; ↓NF-κB; ↓CYP2E1; ↓VEGF; ↓PI3K; ↓Akt; ↓mTOR	10–20 mg/kg (oral)	20 alternate days	Sivalingam et al., 2019 [127]
*Neural cancer*						
Nuciferine	SY5Y cells subcutaneously implanted in nude mice	Reduced tumor weight	Not reported	9.5 mg/kg (i.p.)	3 times a week for 3 weeks	Qi et al., 2016 [96]
Nuciferine	U251 cells subcutaneously inoculated in BALB/c nude mice	Suppressed tumor weight and size	↓Ki-67; ↓CDC2; ↓Bcl-2; ↓HIF1A; ↓N-cadherin; ↓VEGFA	15 mg/kg (i.p.)	Once a day for 2 weeks	Li et al., 2019 [130]
*Skin cancer*						
Procyanidin extract from seedpod	B16 cells inoculated into syngeneic C57BL/6 J mice	Suppressed tumor volume and weight	↓lipid peroxidation levels; ↑SOD; ↑CAT; ↑GSPx; ↑spleen and thymus index	60–120 mg/kg (i.g.)	Every 2–3 days for 15 days	Duan et al., 2010 [137]
Leaf extract	UV-radiation exposed female guinea pigs	Reversed UVB-induced epidermal hyperplasia and hyperpigmentation	↓MITF; ↓tyrosinase; ↓TRP-1; ↓PKA; ↓ERK; ↓melanin	1–2% (topical)	2 weeks	Lai et al., 2020 [138]
7-Hydroxy-dehydronuciferine	A375.S2 cells injected in BALB/c nu/nu female mice	Reduced tumor volume	Not reported	20 mg/kg (i.p.)	Every 7 days for 28 days	Wu et al., 2015 [139]

The authors apologize for any inconvenience caused and state that the scientific conclusions are unaffected. The original article has been updated.

## References

[1] Bishayee A., Patel P.A., Sharma P., Thoutireddy S., Das N. (2022). Lotus (*Nelumbo nucifera* Gaertn.) and Its Bioactive Phytocompounds: A Tribute to Cancer Prevention and Intervention. Cancers.

